# Exposure to lower red to far-red light ratios improve tomato tolerance to salt stress

**DOI:** 10.1186/s12870-018-1310-9

**Published:** 2018-05-24

**Authors:** Kai Cao, Jie Yu, Dawei Xu, Kaiqi Ai, Encai Bao, Zhirong Zou

**Affiliations:** 1 Horticulture College, Northwest A&F University, Yangling, Shaanxi China; 2 The Agriculture Ministry Key Laboratory of Agricultural Engineering in the Middle and Lower Reaches of Yangze River, Nanjing, China; 3 Guangxi Zhong Nong Fu Yu International Agricultural Science and Technology Co., Ltd, Yulin, Guangxi China

**Keywords:** Tomato, R: FR ratio, Phytochrome B1, Antioxidant system, chlorophyll fluorescence

## Abstract

**Background:**

Red (R) and far-red (FR) light distinctly influence phytochrome-mediated initial tomato growth and development, and more recent evidence indicates that these spectra also modulate responses to a multitude of abiotic and biotic stresses. This research investigated whether different R: FR values affect tomato growth response and salinity tolerance. Tomato seedlings were exposed to different R: FR conditions (7.4, 1.2 and 0.8) under salinity stress (100 mM NaCl), and evaluated for their growth, biochemical changes, active reactive oxygen species (ROS) and ROS scavenging enzymes, pigments, rate of photosynthesis, and chlorophyll fluorescence.

**Results:**

The results showed that under conditions of salinity, tomato seedlings subjected to a lower R: FR value (0.8) significantly increased both their growth, proline content, chlorophyll content and net photosynthesis rate (Pn), while they decreased malondialdehyde (MDA) compared to the higher R: FR value (7.4). Under conditions of salinity, the lower R: FR value caused a decrease in both the superoxide anion (O_2_^•−^) and in hydrogen peroxide (H_2_O_2_) generation, an increase in the activities of superoxidase dismutase (SOD, EC 1.15.1.1), peroxidase (POD, EC 1.11.1.7) and catalase (CAT, EC 1.11.1.7). Tomato seedlings grown under the lower R: FR value and conditions of salinity showed a higher actual quantum yield of photosynthesis (ΦPSII), electron transport rate (ETR), and photochemical quenching (qP) than those exposed to a higher R: FR, indicating overall healthier growth. However, the salinity tolerance induced at the lower R: FR condition disappeared in the tomato *phyB1* mutant.

**Conlusion:**

These results suggest that growing tomato with a lower R: FR value could improve seedlings’ salinity tolerance, and phytochrome B1 play an very important role in this process. Therefore, different qualities of light can be used to efficiently develop abiotic stress tolerance in tomato cultivation.

## Background

Plant growth and development are controlled by various signaling pathways that enable them to modulate a wide range of molecular and biochemical responses to changes in their environment. Light is one of the most important environmental factors for plant development. To adapt to different light conditions, plants evolved several families of photoreceptors covering both the visible and the UV-A/B region of the spectrum [1–5].

Phytochromes, which absorb red (R) and far-red (FR) light, are the most characterized photoreceptors in plants and are important in mediating many aspects of physiological development. Phytochromes, composed of photochromic proteins, are ~ 130 kDa peptides with a covalently linked linear tetrapyrrole bilin chromophore, that exist as two photo-interconvertible isomeric forms: the red-light-absorbing form (Pr), which is biologically inactive, and the far-red-light-absorbing form (Pfr), which is biologically active [6]. Upon excitation by R or FR light (producing a high or low R: FR ratio, respectively), the phytochrome converts the Pr into the Pfr form, or vice versa [2, 6].The conversion between Pr and PFr synchronizes plant development with the light environment, which causes changes in the expression of genes involved in photomorphogenesis [7–9].

Besides regulating photomorphogenesis, phytochromes also play an essential role in adapting to different sources of abiotic plant stress [2, 10–15]. The tomato phytochromes B1 and B2 mainly act as negative regulators of growth, pigment maintenance and osmoprotectant accumulation during responses to the different abiotic stresses. However, *phyA* mutant showed similar growth variations under different abiotic stresses when compared to the wild genotype [10]. Indorf et al. [12] found that *phyA*, *phyB* and *phyAphyB Arabidopsis thaliana* mutants showed a reduced expression of salt tolerance genes, and the expression of these genes were also altered by exposure to different light conditions, suggesting that the phytochrome family contributes to salinity stress responses. Compared with the wild type, rice *phyB* deficiency causes both reduced total leaf area and reduced transpiration per unit leaf area, which reduced water loss and improved drought tolerance of *phyB* mutants [16]. In fact, *phyB* seems to be a fundamental component of many plants responses to abiotic stressors.

Soil salinity is a major threat to global food security. During salinity stress, plants have evolved a complex survival response that involves the coordinated action of many physiological and genetic processes, including control of water loss through stomata, ion sequestration, metabolic adjustment, osmotic adjustment, and antioxidative defense [17–20]. The raised level of reactive oxygen species (ROS) under salinity stress such as superoxide (O_2_^•−^) and OH^•^ radicals, peroxynitrite, and H_2_O_2_, and the failure of ROS-scavenging mechanisms leads to oxidative stress, damage to macromolecules, and eventually cell death [18, 21]. The increasing activity of antioxidant enzymes (SOD, CAT, POD, etc.) correlate with the level of salt tolerance [22, 23]. Moreover, salt tolerance can be improved when some antioxidant enzymes are overexpressed [24, 25]. Salinity tolerant hyperactive phytochrome mutant overexpression lines are associated with decreased H_2_O_2_ levels and significantly increased enzymatic activities of the major ROS scavengers, when compared with the wild type [26].

Chlorophyll’s concentration and composition directly influence photosynthetic rate. The effects of environmental stresses on chlorophyll metabolism such as salt stress, light wavelength, and metals, have been studied in plants [27, 28]. There is an observed salinity-induced decrease in chlorophyll, which may be due to a decrease in 5-aminolaevulinic acid accumulation [27]. Knowing the R: FR ratio can provide information about the shade, daylight, and seasonal environment that a plant was grown under, via information about the associated regulation of leaf chlorophyll synthesis, photosynthesis rate, and PSII electron transport [2, 26, 29].

Previous studies have indicated that a well-organized interaction exists between phytochrome and abiotic stresses in plants. However, the exact relationship between the R: FR value and salinity stress on chlorophyll synthesis, photosynthesis rate, and PSII electron transport in the tomato is still elusive. In the present study, the effect of different R: FR values and salinity on tomato seedling growth, biochemicals, ROS and ROS scavenging enzymes, pigment, photosynthesis, and chlorophyll fluorescence is clarified. By clarifying the interaction between R:FR and salinity, we may be able to produce varieties of tomato and other vital food crops that are able to better tolerate and survive increasingly saline soils in diverse environments.

## Results

### Lower R: FR promoted tomato seedling growth under salinity stress

Data showing the effect of a range of R: FR conditions on the growth parameters of tomato seedlings under salinity stress are in Table 1. In general, salinity caused a significant reduction in plant height, stem diameter, fresh and dry weight of root, stem and leaf, as compared to control plants in the same light conditions. Under normal conditions with no salinity in T3, there was a substantial increase in plant height (42.70%), fresh and dry weight (38.51 and 25.59%, respectively), as compared to T1, whereas in T2, there was a larger increase of 23.87, 47.17 and 39.29% respectively, when compared with T1 (Table 1). Under the salinity condition, in T6, the lower R: FR led to a substantial increase in plant height (49.53%), fresh and dry weight (93.17 and 104.88%, respectively), whereas in T5 there was a lower increase of 22.14, 44.95 and 50.00%, respectively, when compared with T3 (Table 1).

**Table 1:** Effects of different R: FR values on plant height, stem diameter, fresh and dry weights of root, stem, and leaf in tomato seedlings under salinity stress in wild type (WT) and phytochrome B1 mutant (*phyB1* mutant)

Treatments			T1	T2	T3	T4	T5	T6
WT	Plant height (cm)		18.43 ± 0.65c	22.83 ± 0.82b	26.30 ± 0.75a	12.92 ± 0.55e	15.78 ± 0.78d	19.32 ± 0.65c
	Stem Diameter (mm)		7.37 ± 0.55a	6.29 ± 0.46b	5.89 ± 0.50bc	5.66 ± 0.55c	5.43 ± 0.53c	4.61 ± 0.17d
	Fresh mass (g)	Root	5.66 ± 0.22b	6.91 ± 0.41a	5.77 ± 0.40b	3.00 ± 0.20d	4.33 ± 0.35c	5.58 ± 0.30b
		Stem	6.38 ± 0.25c	9.57 ± 0.34a	10.94 ± 0.34a	2.73 ± 0.16e	4.47 ± 0.28d	5.89 ± 0.29c
		Leaf	14.44 ± 0.51c	22.49 ± 0.55a	19.96 ± 0.47b	7.43 ± 0.37e	10.29 ± 0.47d	13.96 ± 0.55c
		In total	26.48 + 0.97c	38.97 + 0.86a	36.68 + 1.08b	13.17 + 0.90e	19.09 + 1.06d	25.44 + 1.12c
	Dry mass (g)	Root	0.28 ± 0.02bc	0.37 ± 0.02a	0.30 ± 0.03b	0.14 ± 0.02e	0.20 ± 0.02d	0.26 ± 0.01c
		Stem	0.23 ± 0.02c	0.35 ± 0.02b	0.41 ± 0.02a	0.16 ± 0.03d	0.24 ± 0.02c	0.25 ± 0.02c
		Leaf	1.16 ± 0.05c	1.61 ± 0.04a	1.40 ± 0.03b	0.51 ± 0.04e	0.78 ± 0.04d	1.13 ± 0.04c
		In total	1.68 + 0.07c	2.34 + 0.06a	2.11 + 0.04b	0.82 + 0.08e	1.23 + 0.08d	1.68 + 0.02c
*phyB1* mutant	Plant height (cm)		25.18 ± 0.79a	24.90 ± 0.74a	25.21 ± 0.51a	16.64 ± 0.51b	16.10 ± 0.55b	16.61 ± 0.67b
	Stem Diameter (mm)		5.61 ± 0.23a	5.66 ± 0.23a	5.50 ± 0.13a	4.44 ± 0.25b	4.49 ± 0.30b	4.30 ± 0.20b
	Fresh mass (g)	Root	2.52 ± 0.10a	2.66 ± 0.13a	2.49 ± 0.15a	1.68 ± 0.04b	1.65 ± 0.14b	1.70 ± 0.06b
		Stem	6.37 ± 0.35a	6.39 ± 0.24a	6.33 ± 0.25a	3.59 ± 0.17b	3.51 ± 0.21b	3.42 ± 0.14b
		Leaf	14.47 ± 0.55a	14.41 ± 0.45a	14.12 ± 0.53a	7.94 ± 0.78b	8.17 ± 0.17b	7.96 ± 0.07b
		In total	23.36 ± 0.47a	23.47 ± 0.16a	22.95 ± 0.24a	13.21 ± 0.58b	13.33 ± 0.10b	13.09 ± 0.16b
	Dry mass (g)	Root	0.138 ± 0.005a	0.142 ± 0.006a	0.141 ± 0.015a	0.087 ± 0.006b	0.089 ± 0.010b	0.093 ± 0.015b
		Stem	0.254 ± 0.017a	0.262 ± 0.026a	0.265 ± 0.015a	0.126 ± 0.017b	0.123 ± 0.005b	0.128 ± 0.010b
		Leaf	1.133 ± 0.083a	1.126 ± 0.101a	1.066 ± 0.100a	0.716 ± 0.011b	0.773 ± 0.015b	0.763 ± 0.057b
		In total	1.516 ± 0.083a	1.533 ± 0.100a	1.473 ± 0.120a	0.920 ± 0.017b	0.980 ± 0.010b	0.971 ± 0.072b

The values (mean ± SE, *n* = 6) with different letter within columns are statistically different (*P* ≤ 0.05) according to Duncan’s multiple range test. T1, 0 mM NaCl + 7.4 R: FR; T2, 0 mM NaCl + 1.2 R: FR; T3, 0 mM NaCl + 0.8 R: FR; T4, 100 mM NaCl + 7.4 R: FR; T5, 100 mM NaCl + 1.2 R: FR; T6, 100 mM NaCl + 0.8 R: FR

Under normal conditions, the growth of tomato seedlings was better with an R: FR of 1.2, compared to a higher ratio of 7.4. However, under the salinity condition, the lower R: FR of 0.8 provided tomato seedlings with greater salinity tolerance.

### A lower R: FR exhibited higher soluble protein and proline, while MDA amount and electrolytic leakage decreased under the salinity condition

**Fig. 1:**
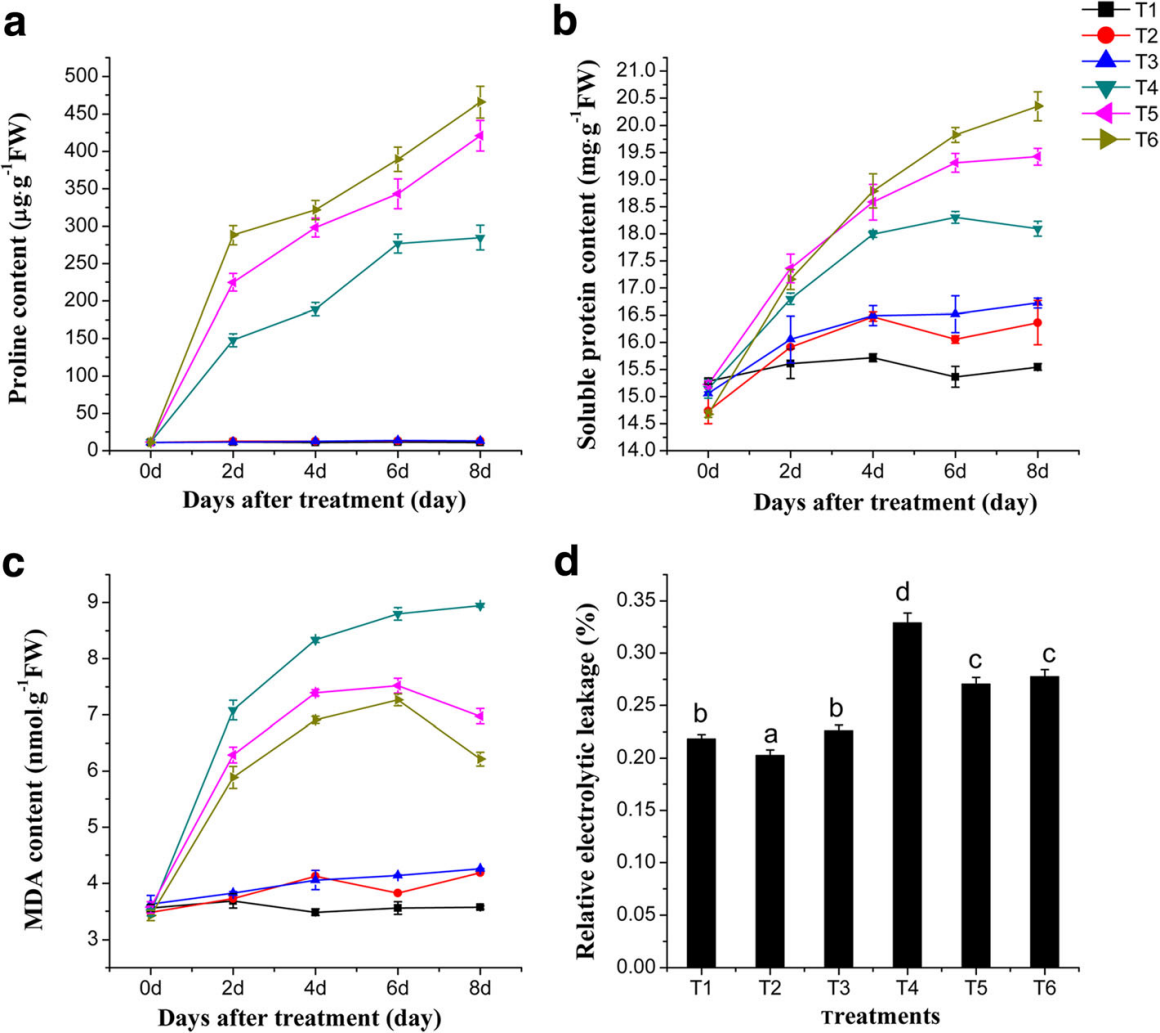
Effects of different R: FR values on proline, soluble protein, and MDA content, and relative electrolyte leakage in tomato seedlings under salinity stress. **a** proline content. **b** soluble protein content. **c** MDA content. Proline, soluble protein and MDA contents were measured 0, 2, 4, 6, and 8 days after exposure to the salinity treatment. **d** relative electrolyte leakage, measured 8 days after salinity treatment. T1, 0 mM NaCl + 7.4 R: FR; T2, 0 mM NaCl + 1.2 R: FR; T3, 0 mM NaCl + 0.8 R: FR; T4, 100 mM NaCl + 7.4 R: FR; T5, 100 mM NaCl + 1.2 R: FR; T6, 100 mM NaCl + 0.8 R: FR. Vertical bars on the lines represent the SE (*n* = 5), Bars with different letters are significantly different at the 0.05 level (Duncan’s multiple range test)

To quantify the effect of salinity stress treatments on the biochemistry of tomato seedlings under different R: FR conditions, the soluble protein, proline, MDA and electrolytic leakage were analyzed. An increased accumulation of proline and soluble protein in plant tissues subjected to stress indicates an effective plant stress response at the metabolic level [26]. It was found that proline and soluble protein accumulated in the greatest quantities under the salinity condition and the lower R: FR of 0.8. After 8 days with the salinity treatment, the amount of proline and soluble protein in the leaf tissues increased by 63.58 and 12.50% under the lower R: FR treatment as compared to the highest ratio tested (Fig. 1a, b). The amount of MDA and the percentage of electrolytic leakage are usually used as tools to assess the severity of oxidative damage, the degree of plant sensitivity, and cell membrane injury [28, 30]. Figure 1 showed that the lower R: FR condition largely decreased MDA and electrolytic leakage percentage under salinity stress. After 8 days of salinity treatment under the lower R: FR, the amount of MDA and electrolytic leakage percentage in the leaf tissue decreased by 30.53 and 15.60%, compared with the highest R: FR condition (Fig. 1c, d). Under the salinity condition, the lower R: FR ratio led to decrease in both MDA amount and electrolytic leakage, but increase in proline amount and accumulation of soluble protein, which made tomato seedlings more tolerant to salinity stress.

### The lower R: FR treatment accumulated less ROS and showed a higher activity of ROS scavenging enzymes under the salinity condition

**Fig. 2:**
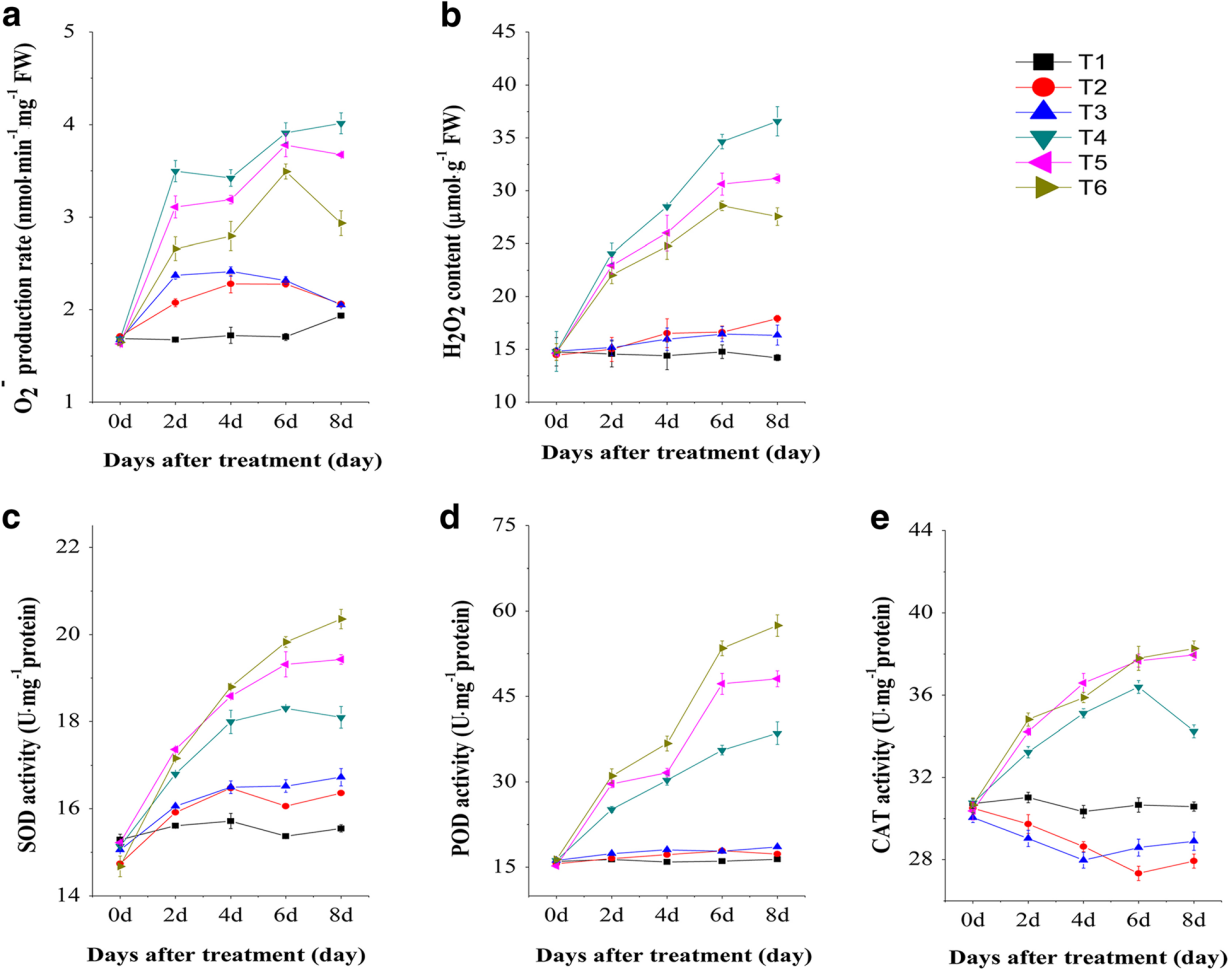
Effects of different R: FR values on on ROS (O_2_^•−^ and H_2_O_2_) and ROS scavenging enzymes (SOD, POD and CAT) activities in tomato seedlings under salinity stress. **a** O_2_^•−^ content. **b** H_2_O_2_ content. **c** SOD activity. **d** POD activity. **e** CAT activity.These parameters were measured 0, 2, 4, 6, 8 days after exposure to salinity treatment. T1, 0 mM NaCl + 7.4 R: FR; T2, 0 mM NaCl + 1.2 R: FR; T3, 0 mM NaCl + 0.8 R: FR; T4, 100 mM NaCl + 7.4 R: FR; T5, 100 mM NaCl + 1.2 R: FR; T6, 100 mM NaCl + 0.8 R: FR.Vertical bars on the lines represent the SE (*n* = 5)

To observe the effect of salinity stress treatments on plant antioxidant system under the lower R: FR condition, H_2_O_2_ and O_2_^•−^ levels and enzymatic activities of the major ROS scavengers were measured. H_2_O_2_ and O_2_^•−^ are common ROS produced in plants, and are indicators of optimal health, as they are typically produced following exposure to salinity stress [31, 32]. In this experiment, the accumulation of H_2_O_2_ and O_2_^•−^ were significantly enhanced after salinity treatment, and after 8 days, the seedlings grown under the higher R: FR treatment showed almost 1.3–1.5-fold higher levels of H_2_O_2_ and O_2_^•−^ than the corresponding tomato seedlings grown under the lower R: FR treatment (Fig. 2a, b). The activity of SOD, POD and CAT enzymes were notably increased in response to salinity alone, but in combination with the lower R: FR treatment, enzyme activity was comparatively much higher (Fig. 2c, d, e). The activity of major ROS-scavenging enzymes revealed that tomato seedlings grown under the lower R: FR treatment exhibited an improved ROS scavenging system under salinity stress compared to that of tomato seedlings grown under the higher R: FR treatment.

### The lower R: FR treatment accumulated more photosynthetic pigments and exhibited increased photosynthetic efficiency

**Fig. 3:**
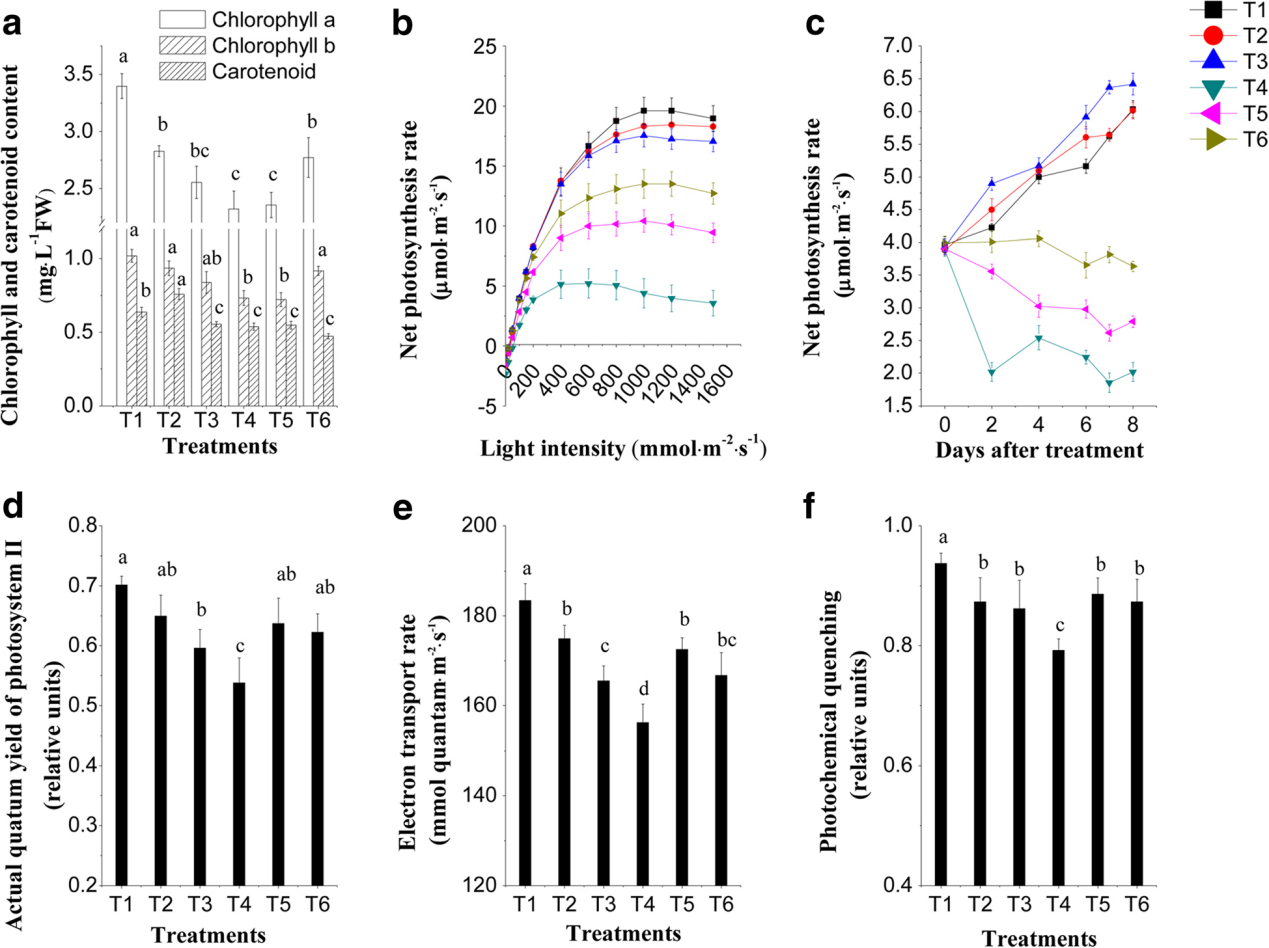
Effects of different R: FR values on chlorophyll content, photosynthesis rate, and PSII electron transport in tomato seedlings under salinity stress. **a** chlorophyll a, chlorophyll b and carotenoid content. **b** light responsive curve. **c** net photosynthesis rate. **d** actual photochemical efficiency of PSII. **e** electron transport rate. **f** the photochemical quenching coefficient. Chlorophyll content, the light responsive curve and PSII electron transport parameters were measured eight days after salinity treatment. Net photosynthesis rate was measured 0, 2, 4, 6, and 8 days after exposure to salinity treatment. T1, 0 mM NaCl + 7.4 R: FR; T2, 0 mM NaCl + 1.2 R: FR; T3, 0 mM NaCl + 0.8 R: FR; T4, 100 mM NaCl + 7.4 R: FR; T5, 100 mM NaCl + 1.2 R: FR; T6, 100 mM NaCl + 0.8 R: FR. Vertical bars on the lines represent the SE (n = 5), Bars with different letters are significantly different at the 0.05 level (Duncan’s multiple range test)

Measurements of chlorophyll, carotenoid, photosynthesis rate, and fluorescence were taken using leaf number two below the youngest fully expanded leaf. Under normal conditions, chlorophyll a had a tendency to decrease with the decrease of R: FR values, no significant differences in chlorophyll b and net photosynthesis rate were observed under different R: FR values, and carotenoid showed a significant difference, not correlated to the R: FR values (Fig. 3a, b, c). However, after the salinity treatment, chlorophyll a, chlorophyll b, and the net photosynthesis rate increased significantly when R: FR value is 0.8, compared with R: FR value is 7.4 (Fig. 3a, b, c). The actual quantum yield of photosynthesis (ΦPSII), electron transport rate (ETR), and photochemical quenching (qP) are inversely proportional to the damage in the PSII reaction centers [31, 32]. These widely used chlorophyll fluorescence parameters indicate how well a plant grows under conditions when higher than normal salinity is present. In the present study, a similar trend was also observed with ΦPSII, ETR, and qP, in which these parameters increased substantially under the lower R: FR treatment (Fig. 3d, e, f), suggesting that a lower R: FR could activate PSII reaction centers and consequently alleviate negative impacts of salinity stress on tomato seedlings.

### Phytochrome B1 is involved in the regulation of lower R: FR on tomato seedlings salinity tolerance

**Fig. 4:**
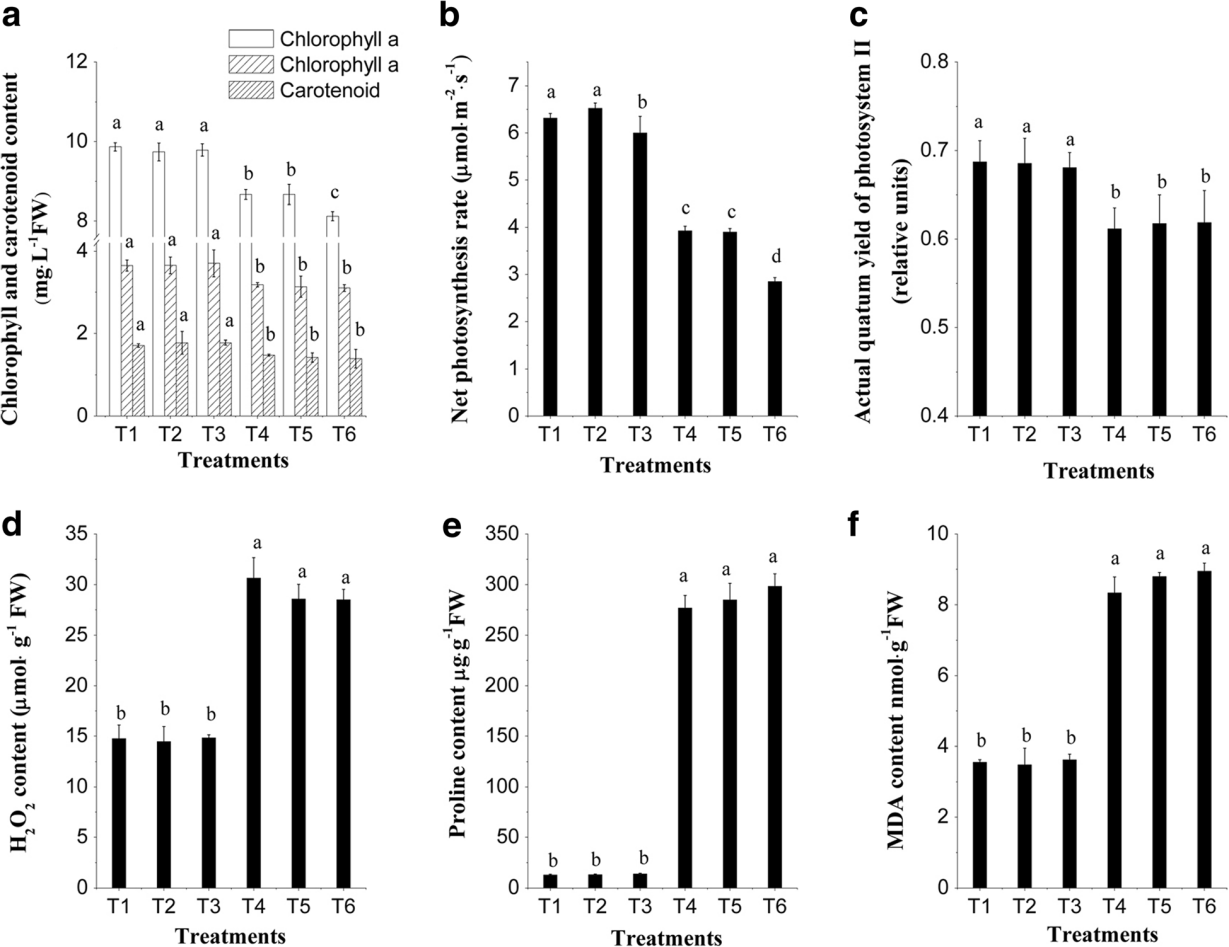
Effects of different R: FR values on on chlorophyll content, photosynthesis rate, actual photochemical efficiency of PSII, and the content of H_2_O_2_, proline and MDA in tomato *phyB1* mutants under salinity stress. **a** chlorophyll a, chlorophyll b and carotenoid content. **b** net photosynthesis rate. **c** actual photochemical efficiency of PSII. **d** H_2_O_2_ content. **e** proline content. **f** MDA content. All these parameters were measured 8 days after salinity treatment. T1, 0 mM NaCl + 7.4 R: FR; T2, 0 mM NaCl + 1.2 R: FR; T3, 0 mM NaCl + 0.8 R: FR; T4, 100 mM NaCl + 7.4 R: FR; T5, 100 mM NaCl + 1.2 R: FR; T6, 100 mM NaCl + 0.8 R: FR. Vertical bars on the lines represent the SE (n = 5), Bars with different letters are significantly different at the 0.05 level (Duncan’s multiple range test)

When phytochrome B1 activity was quantified to test for its influence on tomato salinity tolerance, results showed that in the tomato *phyB1* mutant, the salinity treatment decreased plant height, stem diameter, fresh and dry weight of root, stem and leaf, chlorophyll and carotenoid amount, photosynthesis rate, ΦPSII and MDA, and increased proline and H_2_O_2_ content, but these parameters did not significantly change when different R: FR treatments were applied under salinity conditions (Table 1, Fig.4). In wild type tomato, different R: FR treatments had a significant influence on these parameters under salinity conditions, however, the influence disappeared in the *phyB1* mutant. These results showed that phytochrome B1 mediated tomato seedlings tolerate high salinity conditions under different R: FR treatments.

## Discussion

Red (R) and far-red (FR) light are abiotic factors that are particularly important for plant growth and development. The ratio of red to far-red light (R: FR) is often used to signal the proximity of neighboring or canopy vegetation, as chlorophyll selectively absorbs 655–665 nm light while transmitting 725–735 nm light. The ratio can also be used to sense changes in daylength or seasonal variation, as the R: FR is low (about 0.6–0.8) at the beginning and end of the photoperiod, and at its maximum at solar noon (about 1.0 to 1.3) [33, 34]. The R: FR value can trigger many plant morphological responses, including promotion of shoot elongation and reduction of stem diameter in dicotyledonous and ornamental species from a low R: FR, either during the daytime or only at the end of the day [29, 35]. During salinity-induced stress, plants experience reduced growth and development, but can respond in various ways to withstand the stress. In this study, without salinity treatment, plant height was increased, but stem diameter were comparatively reduced under a lower R: FR treatment (Table 1). Similarly, Yan et al. [36] reported that soybean seedling height was significantly increased while the stem diameter was decreased in lower R: FR treatment conditions. However, under a salinity treatment, applying a lower R: FR promoted tomato seedlings growth, increased plant height, fresh and dry weight of root, stem and leaf. Results from this research suggest that a lower R: FR condition could help alleviate the impact of salinity on the development of tomato seedlings, and that more generally, the value of R: FR influences salinity stress tolerance as a consequence of morphological responses. To better quantify and operationalize this finding, further studies on the relationship of salinity tolerance with light quality are necessary.

A given plant’s ability to tolerate salinity is dependent on multiple biochemical pathways that lead to the production of osmotically dynamic metabolites, free radicals, and specific proteins that manage ion and water flux [28, 37]. Likewise, the R: FR ratio also regulates a large range of biochemical processes throughout a plant’s life [29]. Proline plays a crucial role in stabilizing the subcellular structures and scavenging free radicals [26]. MDA is the decomposition product of polyunsaturated fatty acids of biomembranes and is used to assess the severity of oxidative damage [28]. Electrolyte leakage is an important index of the plant cell’s permeability, which plays an important role in the study of plant salt stress [30]. Our results indicated that a lower R: FR of 0.8 under salinity conditions led to a decrease in MDA and electrolytic leakage, and an increase in proline and soluble protein content (Fig. 1). These results suggest that salt-stressed tomato seedlings grown under lower R: FR conditions encountered less cellular damage and lipid peroxidation.

H_2_O_2_ and O_2_^•−^ are reactive oxygen species (ROS), and cause oxidative stress at high concentrations [21]. In this study, accumulation of H_2_O_2_ and O_2_^•−^ from salinity stress was decreased when seedlings were exposed to a lower R: FR condition. Plant cells have developed different mechanisms to alleviate the excess ROS, and keep the balance of the formation and removal of ROS [38]. An increase in antioxidant enzymes (SOD, POD and CAT) protects the plants from oxidative damage, and were shown to be highest in the lower R: FR condition under salinity, suggesting that the lower R: FR condition induced an efficient ROS scavenging mechanism. Similar findings of low accumulation of ROS and higher antioxidant enzyme activity in stress-tolerant plants have been reported previously [26, 28]. Although the mechanism of light quality regulated ROS generation and scavenging remains unclear, there are reports indicating that light quality induces the synthesis of various protective compounds, including antioxidant enzymes and protective proteins [39, 40].

Salinity is known to influence chlorophyll synthesis and photosynthesis in a number of plant species, and chlorophyll content tends to positively correlate with photosynthetic rate [31, 32]. Previous studies have found that salinity stress and a lower R: FR value inhibits the biosynthesis of chlorophyll [29, 37]. However, in our study, the upregulation of Chl a, Chl b, and the net photosynthesis rate were observed in tomato leaves under salinity when exposed to a lower R: FR condition (Fig. 3). To explain these results, further studies on the interaction between salinity and the R: FR value on chlorophyll biosynthesis need to be performed. Maybe salinity and the R: FR value influence chlorophyll biosynthesis in different ways, and a lower R: FR value may help protect chlorophyll biosynthetic enzymes or products that were damaged by salinity. Wang et al. [41] reported that lower R: FR light conditions significantly alleviate PSII and PSI photoinhibition in the shade leaves of tomato plants, and lower R: FR illumination induced nonphotochemical quenching of chlorophyll a fluorescence and increased the activities of Foyer-Halliwell-Asada cycle enzymes and cyclic electron flux (CEF) around PSI. The parameters of the actual quantum yield of photosynthesis (ΦPSII), electron transport rate (ETR), and photochemical quenching (qP) have been used extensively to examine the photosynthetic efficiency of different crop plants subjected to salinity stress [32]. Under salinity, the highest ΦPSII, ETR, and qP values were observed in the lower R: FR condition, suggesting that a lower R: FR condition alleviated the salinity-induced inhibition of PSII electron transport, allowing tomato seedlings to effectively tolerate the stress. These results are in agreement with those of Shu et al. [32], who reported that exogenous materials could relieve salinity-induced inhibition of electron transport at the acceptor side of the PSII reaction center.

Phytochromes are involved in plant tolerate to biotic and abiotic stressors [10, 26, 29, 42]. Tomato contains five phytochrome genes, named PHYA, PHYB1, PHYB2, PHYE and PHYF [43]. In tomato, PHYB1 is mainly involved in the response to R and FR light, and controls seedling hypocotyl elongation, anthocyanin accumulation, cotyledon expansion, flowering, and abiotic tolerance [10, 44]. This study demonstrated that different values of R: FR had a significant influence on the salinity tolerance of wild type tomato seedlings, however, some of the influence disappeared in the tomato *phyB1* mutants. In the tomato *phyB1* mutant, plant height, stem diameter, fresh and dry weight of root, stem and leaf, chlorophyll b and carotenoid amount, ΦPSII, MDA, proline, and H_2_O_2_ content did not significantly change when different R: FR treatments were applied under salinity conditions. However, these parameters significantly changed in wild type tomato seedlings after lower R: FR treatments under salinity conditions. These results suggest that a lower R: FR condition improved tomato salinity stress tolerance, and phytochrome B1 play an very important role in this process. Although the role of phytochromes under abiotic stress conditions is largely unknown, there are many reports indicating that phytochromes might mediate the abiotic stress response in plants to control antioxidant enzymes like peroxidases and non-enzymatic antioxidants such as ascorbate, carotenoids, and flavonoids under stress conditions [10, 42]. Plants exposed to light with different R: FR values have been reported to control phytohormone biosynthesis (gibberellin, auxin, cytokinins and abscisic acid), which are also involved in plant salinity tolerance [2, 45]. Furthermore, phytochrome regulates the expression of some proteins that mediate salt tolerance [12]. These findings suggested a strong correlation between phytochromes and salinity tolerance that might involve a complex signaling network which has yet to be elucidated.

## Conclusion

This study identified the effect of R: FR values on tomato salinity tolerance. The results suggested that lower R: FR values could significantly alleviate salt-induced oxidative damage on tomato seedlings, most likely through regulation of antioxidant enzymes and non-enzymatic systems, and phytochrome B1 play an very important role in this process. This was associated with an improvement in the PSII electron transport and promoted tomato seedlings growth under salinity conditions. These findings also indicate possible opportunities to explore the relationship between light quality and salinity stress while also supporting future environmental attempts to improve tolerance to salinity stresses in other crops.

## Methods

### Plant material and growth conditions

In this study, cv. MoneyMaker (*Solanum lycopersicum* L.) wild type and MoneyMaker backgrounded phytochrome B1 mutant (*phyB1*) were used as the experimental organism. The mutant was provided by the Tomato Genetic Resource Center (Department of Vegetable Crops, University of California, Davis), TGR accession number LA4357. Tomato seeds were soaked for 30 min in 50% bleach, then rinsed in running water thoroughly and directly sown on germination paper and incubated at 25 °C. After germination, seedlings were sowed into commercial substrate on polystyrene plugs (plug size: 50 cm × 25 cm × 4.5 cm; 50 cavities very plug, one seed per cavity). Seedlings were grown in a growth chamber under day-neutral conditions (12 h light/12 h dark), maintained constantly at 24–26 °C with 40–45% relative humidity. When the second leaf fully expanded, the same sized seedlings were selected and washed to remove the commercial substrate, and then transplanted on foam plates to be grown hydroponically in the growth chamber (Northwest A&F University, Yangling, Shaanxi, China). The growth chamber was an airtight space, equipped with a lighting system and air conditioner cooling and heating system.

The growth chamber was maintained at 29–31 °C during the day with 70–80% relative humidity, and 24–26 °C during the night with 40–50% relative humidity (12 h light/12 h dark). The growth chamber had six sections, divided by an opaque silvery cloth, with 36 seedlings in each section. The growth chamber used light-emitting diodes with an intensity of 200–230 μmol·m^− 2^·s^− 1^. The seedlings were grown with Yamasaki tomato nutrient solution at 0.5 concentration (pH 6.0–6.5, EC: 1.3–1.5 ms·cm^− 1^), with dissolved oxygen maintained by air pump at 80 ± 0.2 mg·L^− 1^.

### Salinity and R: FR light treatment

**Fig. 5:**
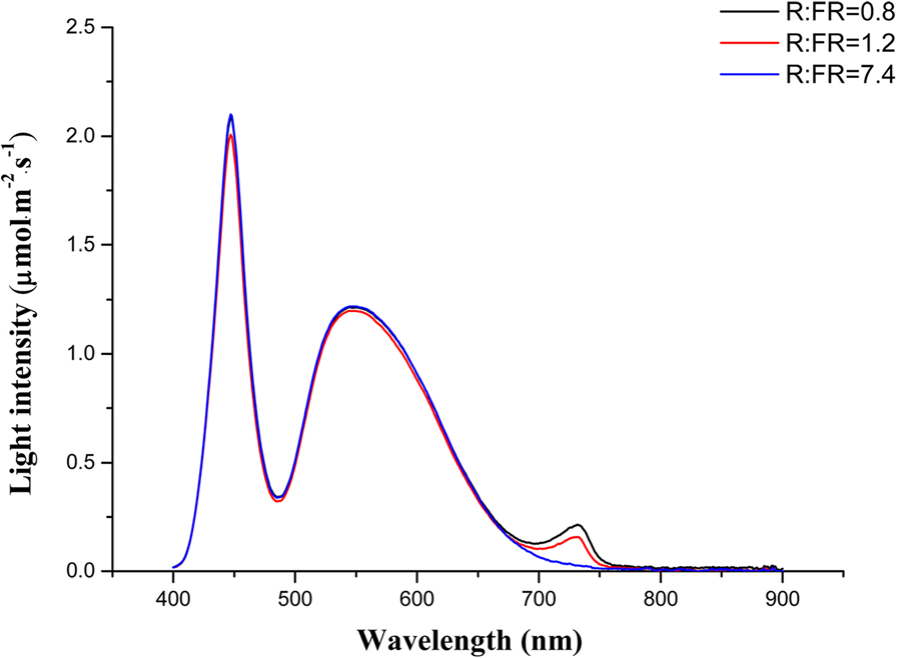
Spectral distribution characteristics of white and FR LED used for different R: FR treatments. The black curve represents R: FR value is 0.8, the red curves represents R: FR value is 1.2; blue curve represents R: FR value is 7.4

After 3 days of pre-culture under normal conditions, treatments with salt (NaCl) and three different R: FR were applied. We use white LED (peaked at 455 and 570 nm) and FR LED (peaked at 730 nm) to justify tomato growth light environment. The experimental plots included six treatments: ① T1, 0 mM NaCl + 7.4 R: FR, ② T2, 0 mM NaCl + 1.2 R: FR, ③ T3, 0 mM NaCl + 0.8 R: FR, ④ T4, 100 mM NaCl + 7.4 R: FR, ⑤ T5, 100 mM NaCl + 1.2 R: FR, ⑥ T6, 100 mM NaCl + 0.8 R: FR. The light intensity and spectrum were measured by a spectroradiometer (PAR-NIR, Apogee Instruments Inc., Logan, UT) and are shown in Fig. 5. For the purposes of this experiment, the R: FR = (photon irradiance between 655 and 665 nm) / (photon irradiance between 725 and 735 nm). The nutrient solutions were renewed every 2 days. After 8d of salinity treatment, the tomato seedlings growth parameters were measured. Tomato wild type samplings were carried out 0, 2, 4, 6 and 8 d after treatments, phyB1 mutant samples were carried out 8 d after treatments, and rapidly frozen these samples in liquid nitrogen and stored at − 80 °C for biochemical analysis, and 3 replicates were used for each analysis.

### Determination of growth parameters

Ten tomato seedlings in each treatment was used to measure the growth parameters. Stem height was measured by a ruler and stem diameter was measured using digital calipers (Digimatic Caliper; Shengli Co., Ltd., Beijing, China). To determine the fresh weight of the root, stem, and leaf, plants were harvested separately, washed with sterile distilled water, and weighed by a balance. The dry weight of plant root, stem, and leaf were determined after drying at 70 °C for 2 days and weighed by a balance.

### Determination of proline content

The concentration of proline was prepared and estimated following the method of Gururani et al. [11]. From each treatment, tomato leaf (0.2 g) was homogenized in 2 mL of 3% (*w*/*v*) sulfosalicylic acid, then centrifuged (15,000 g, 10 min, 25 °C). Then, 0.5 mL glacial acetic acid and 0.5 mL 2.5% ninhydrin solution were added into 0.5 mL supernatant. The mixture was heated at 100 °C for 30 min in a water bath, cooled in an ice bath, then added to 1.5 mL toluene and allowed to rest for at least 10 min to allow the phases to separate. The absorbance of the toluene fraction was read at 520 nm. The proline content was determined by a calibration curve operated in a similar way with solutions of proline at different concentrations.

### Determination of malondialdehyde (MDA) content

Oxidative damage to lipids was estimated by measuring the content of MDA. One gram of plant material from each treatment was homogenized with 5 mL 5% (w/v) trichloroacetic acid (TCA), then centrifuged (15,000 g, 15 min, 4 °C) and added to 5% trichloroacetic acid containing 0.65% (w/v) 2-thiobarbituric acid (TBA) in 2 mL supernatant. The mixture was heated at 100 °C for 15 min in a water bath and then quickly cooled on ice, then the contents were centrifuged (15,000 g, 15 min, 4 °C) and the absorbance was read at 532 nm, as described by Huang et al. [28].

### Determination of electrolyte leakage

The total inorganic ions that leaked out of the leaves were measured by the method described by Abo-Ogiala et al. [17]. Ten leaf disks were cut, weighed, and subsequently placed at room temperature into Falcon tubes containing 10 mL of deionized water. Conductivity of the deionized water (EC_0_) and soaked plant tissue was measured after 24 h (EC_1_) using a conductivity meter (DDSJ-308A, Shanghai Jingke Instrument Co., Ltd., China). To determine the maximum electrolyte leakage (EC_2_), the samples were boiled at 100 °C for 20 min and then cooled at room temperature. The relative electrolyte leakage (%) was calculated as: (EC_1_-EC_0_) / (EC_2_-EC_0_) × 100%.

### Determination of H_2_O_2_ and O_2_^•−^

To determine O_2_^•−^, the assay was done as described by Zhao et al. [46] with some modifications. A section of plant leaf (0.3 g) was homogenized with 3 mL 50 mM phosphate buffer (pH 7.8) and centrifuged (12,000 g, 20 min, 4 °C). One mL of hydroxylamine hydrochloride was added to 1 mL of the supernatant, and the mixture was incubated at 25 °C for 20 min. One mL of 17 mM γ-amino-phenylsulfonic and 1 mL of 7 mM α-amino-phenylsulfonic were added to the mixture for another 20-min incubation at 30 °C. The absorption of the reaction mixture was monitored at 530 nm. O_2_^•−^ was calculated according to a standard curve based on Sodium nitrite.

The concentration of H_2_O_2_ was prepared and estimated following the method of Gururani et al. [26], with slight modifications. Fresh samples of leaf (0.5 g) were homogenized with 5 mL 0.1% (*w*/*v*) TCA in an ice bath, and the homogenate was centrifuged at 12,000 g for 15 min. Then, 0.5 mL of 100 mM potassium phosphate buffer (pH 7.0) and 1 mL of 1 M KI were added to 0.5 mL of the supernatant. The absorbance of supernatant was read at 390 nm, and the content of H_2_O_2_ was calculated based on a standard curve.

### Assay of soluble protein and antioxidant enzymes (SOD, POD, and CAT)

Fresh tomato leaf (0.3 g) from each treatment was crushed into fine powder in a pestle and mortar using liquid nitrogen. The powder was homogenized in 3 mL of 50 mM pre-cooled phosphate buffer (pH 7.8). The homogenate was centrifuged (12,000 g, 20 min, 4 °C), and the supernatant was used for enzyme activity assays. Protein content was evaluated according to the method of Huang et al. [28], using bovine serum albumin as the standard.

Total SOD activity was determined by the inhibition of the photochemical reduction of nitroblue tetrazolium (NBT), as described by Moradi and Ismail [31], with slight modifications. The reaction mixture consisted of 0.1 mL of enzyme extract and 5 mL O_2_^•−^ generating solution which contained 4 mL 14.5 mM methionine, 0.02 mL 30 μM EDTA-Na_2_, 0.4 mL 750 μM NBT, and 0.4 mL 20 μM of riboflavin. Extracts were brought to a final volume of 0.18 mL with 50 mM Na-phosphate (pH 7.8). Test tubes were shaken and placed 30 cm under lights for 15-min. The enzyme unit was defined as the inhibition of the photochemical reduction of 50% NBT by the reaction system per minute at 560 nm, using a Multiple-label Multifunctional Microplate reader (spectraMax i3x, Molecular Devices Inc., United States). Enzyme activity was expressed as U·mg^− 1^ protein.

CAT activity was determined by the reduction of H_2_O_2_ at 240 nm spectrophotometrically, as described by Gururani et al. [26], with some modifications. The reaction mixture contained 13.6 mM H_2_O_2_ in 15 mM phosphate buffer (pH 7). The reaction was initiated by the addition of the enzyme extract. POD activity was determined with guaiacol as the reducing substrate in a reaction mixture containing 0.2 M Na-phosphate buffer (pH 6), 3 mM guaiacol, and 4.9 mM H_2_O_2_. The oxidation of guaiacol was assessed by recording the absorbance increase at 470 nm. One unit of CAT and POD was defined as the amount of enzyme which produced a change of 0.1 in absorbance at 240 nm and 470 nm, respectively, per minute, at 25 °C. The enzyme activities of POD and CAT were expressed as U·mg^− 1^ protein.

### Chlorophyll concentration and photosynthetic rate analysis

Tomato leaf (0.3 g) was soaked in 95% alcohol for extraction. The samples were then stored at 25 °C for 24 h. The absorbance was read at 665, 649 and 470 nm. The concentrations of chlorophyll a, chlorophyll b and cartotenoid were calculated according to Fan et al. [27].

Net photosynthesis rate (Pn) and the light responsiveness curve of tomato leaves were determined using the second fully expanded leaves between 9:00 am and 11:00 am on a portable photosynthesis system (LI6400, LI-COR Inc., USA). In the assimilation chamber, leaf temperature was 25 °C, relative humidity was 85%, ambient CO_2_ concentration was 400 ± 10 ppm, and photosynthetic photon flux density (PPFD) was 200 μmol·m^− 2^·s^− 1^.

### Chlorophyll fluorescence parameters

Chlorophyll fluorescence emission from the upper surface of the second fully expanded leaves of intact plants was measured by a modulated fluorimeter (PAM Photosynthesis Yield Analyser, Walz, Effeltrich, Germany), according to Moradi and Ismail [31]. The minimal (Fo) and maximal (Fm) chlorophyll fluorescence emissions were assessed in leaves after 30-min of dark adaptation. To determine the minimal fluorescence level in a leaf during illumination (Fo’), and to allow maximal oxidation of the PSII centers in the presence of far-red light, a black cloth was rapidly placed over the plants to block out light, causing the leaf fluorescence to fall to the Fo’ level, rising again after a few seconds. Then, the leaves were continuously illuminated with a white actinic light, which was equivalent to the actual growth lights used for the tomato plants, to measure both steady-state (Fs) and the maximal chlorophyll fluorescence level in light-adapted leaves (Fm′). From these measurements, the actual photochemical efficiency of PSII [ΦPSII = (Fm′- Fs) / Fm′], the photochemical quenching coefficient [qP = (Fm′- Fs) / (Fm′ − Fo’)], electron transport rate (ETR = ΦPSII ×PPFD × 0.5 × 0.87) were calculated, where PPFD is the photosynthetic photon flux density incident on the leaf, 0.5 is a factor that assumes equal distribution of energy between the two photosystems, and 0.87 is an assumed factor of leaf absorbance.

### Statistical analysis

Statistics were calculated using SPSS 20.0 (SPSS, version 20.0, IBM Inc., USA). The data were analyzed by analysis of variance (ANOVA), and the differences between the means were assessed by Duncan’s multiple range test (*P* < 0.05). Error bars in all figures represent standard deviation from the mean. Graphs were created using OriginPro (version 8.0, Origin Lab, MA, USA).

## Abbreviations

CAT: Catalase
ETR: Electron transport rate
FR: Far-red
H_2_O_2_: Hydrogen peroxide
MDA: Malondialdehyde
O_2_^•−^: Superoxide anion
Pn: Net photosynthesis rate
POD: Peroxidase
PSII: Photosystem II
qP: Photochemical quenching
R: Red
ROS: Reactive oxygen species
SOD: Superoxidase dismutase
ΦPSII: Actual quantum yield of photosynthesis
